# Case report: Creutzfeldt-Jakob disease presenting with anxiety symptoms in a COVID-19 post-infection patient

**DOI:** 10.3389/fneur.2023.1239576

**Published:** 2023-08-07

**Authors:** Christl S. K. Yong, Ethan Jian-Hui Maniam, Cheryl W. L. Chang, Jonathan Yexian Lai, Cyrus Su Hui Ho

**Affiliations:** ^1^Department of Psychological Medicine, National University Health System, Singapore, Singapore; ^2^Yong Loo Lin School of Medicine, National University of Singapore, Singapore, Singapore; ^3^Department of Neurology, National Neuroscience Institute, Duke-NUS Medical School, Singapore, Singapore; ^4^Department of Psychological Medicine, Yong Loo Lin School of Medicine, National University of Singapore, Singapore, Singapore

**Keywords:** case report, Creutzfeldt-Jakob disease, COVID-19, anxiety, inflammation

## Abstract

Creutzfeldt-Jakob Disease (CJD) is a rare, rapidly progressive, and fatal neurodegenerative disorder. We describe a man whose initial manifestations of CJD occurred shortly after contracting Coronavirus disease 2019 (COVID-19). He first developed anxiety and short-term memory loss a few weeks after a mild COVID-19 infection. He subsequently developed parkinsonism, eventually progressed to akinetic mutism, and passed away 5 months after symptom onset. This case highlights a potential temporal relationship between COVID-19 infection and the onset of neurodegenerative symptoms. Microglia and astrocytes in the central nervous system (CNS) and ‘S1’ spike proteins on SARS-CoV-2 are potential mediators in neuroinflammation and neurodegeneration.

## Introduction

Creutzfeldt-Jakob Disease (CJD) is a rare but rapidly progressive and invariably fatal neurodegenerative disorder associated with accumulating misfolded prion protein in the central nervous system (CNS). Patients present with neurocognitive deficits, ataxia, and abnormal movements such as myoclonus, dystonia or chorea (1). They may also exhibit prodromal psychiatric symptoms such as sleep difficulties, psychotic features, agitation and mood disorders (2). CJD comprises three subtypes: sporadic, inherited and acquired. In most individuals, CJD occurs sporadically, and the age of onset is most commonly between 60 and 70 years, with a median survival time of 5 months following symptom onset (3).

Severe acute respiratory syndrome coronavirus (SARS-CoV-2) infection causes coronavirus disease 2019 (COVID-19). To date, COVID-19 has affected millions around the world. The infection frequently presents with fever and respiratory symptoms. However, neuropsychiatric symptoms are also frequently reported in COVID-19 patients, including delirium, neurocognitive disorders and psychiatric disorders such as depression and anxiety (4). Even up to 6 months post COVID-19 infection, there is an increased risk of neurologic and psychiatric morbidity (5).

Herein we describe a case of CJD manifesting itself following COVID-19 infection.

## Case description

We present a case of a 70-year-old man with a past medical history of prostate cancer treated with radiation and hormonal therapy. Notably, he had a family history of motor neurone disease with three siblings who shared the diagnosis.

He was first diagnosed with a mild COVID-19 infection in March 2022. Subsequently, he required admission to our institution, a tertiary medical hospital, following a 3-week history of exertional dyspnoea associated with palpitations, anorexia, weight loss and feelings of anxiety. He reported that these symptoms started soon after COVID-19 infection. A full blood count revealed a hemoglobin count of 8.7 g/dL. He was diagnosed with symptomatic anemia secondary to radiation proctitis and discharged.

He was re-admitted in April 2022 with persisting symptoms of dyspnoea associated with palpitations and insomnia. His mood was not depressed, and he did not exhibit other features suggestive of panic attacks or generalized anxiety. He reported significant health anxiety following his COVID-19 infection and interpersonal difficulties with his wife. The medical evaluation did not reveal any other cause for his symptoms, and a repeat full blood count showed a hemoglobin level of 10 g/dL. He was thus diagnosed with adjustment disorder with anxiety symptoms and treated with sertraline 50 mg at night.

During his outpatient psychiatric appointment in May 2022, he reported ongoing health anxiety over his symptoms of persistent dyspnoea. He was not compliant with sertraline due to a sensation of uneasiness after one dose and was switched to escitalopram 10 mg daily. Additionally, he received outpatient psychological therapy. After starting antidepressant treatment, he reported improvement in anxiety symptoms.

He was admitted 2 months later, in July 2022, for per rectal bleeding from radiation proctitis. During the admission, his wife highlighted symptoms of rapid cognitive decline that began a few weeks following his COVID-19 infection when he exhibited progressive short-term memory issues that included worsening forgetfulness and getting lost in familiar places. His mood was not depressed, there were no psychotic symptoms, and he was oriented to time, place and person. Physical examination revealed hypomimia, shuffling gait and frontal release signs comprising a positive palmomental reflex and glabellar tap. There were no signs suggestive of anterior horn cell disease. His Montreal Cognitive Assessment (MOCA) score was 13/30, revealing deficits in orientation, recall and executive function.

## Diagnostic assessment and follow-up

**Figure 1:**
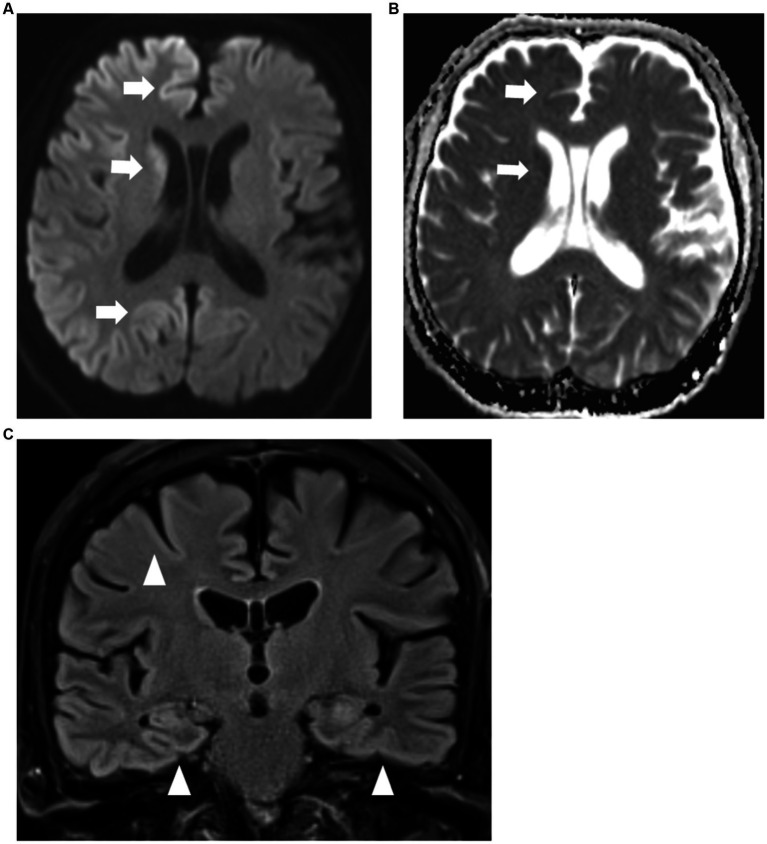
**(A,B)** MRI brain axial diffusion weighted imaging sequence **(A)** with corresponding apparent diffusion co-efficient sequence **(B)** showing restricted diffusion of both cerebral cortices and caudate nuclei (arrows) which are more prominent on the right. **(C)** MRI brain coronal FLAIR sequence showing FLAIR hyperintensities of bilateral cerebral cortices (arrowheads).

His hemoglobin was 6.1 g/dL on admission from per rectal bleeding. He received a blood transfusion and Argon Plasma Coagulation during flexible sigmoidoscopy. His hemoglobin level stabilized during the admission. Thyroid function, liver and renal function were normal. Syphilis and HIV tests were negative. Magnetic Resonance Imaging (MRI) of the brain showed areas of gyriform restricted diffusion with FLAIR hyperintensities involving bilateral cerebral cortices and caudate nuclei (Figures 1A–C).

Electroencephalogram (EEG) showed a right frontotemporal epileptogenic focus with intermittent discharges at a rate of 1 Hz. Cerebrospinal fluid (CSF) analysis showed a white blood cell count of <1/μL, red blood cell count of 3/μL, protein 0.29 g/L (reference range: 0.15–0.40 g/L), glucose 3.9 (reference range 2.2–3.9 mmol/L). Paired serum glucose was 7.0 mmol/L. CSF meningitis and encephalitis DNA & RNA PCR tests were negative. Serum and CSF tests for autoimmune encephalitis and paraneoplastic antibodies were also negative. CSF RT-QUIC returned positive with T tau protein >20,000 pg./mL (reference range: 0–1,149 pg./mL) and 14–3-3 Gamma 69,601 AU/mL (reference range: <30–1999 AU/mL), with an overall likelihood of prion disease >98%. These CSF and radiological findings confirmed the clinical suspicion for sporadic CJD (Table 1).

**Table 1:** Summary of investigations performed.

Investigation	Reference range	Result
Hematological		
White blood cells	4.30–10.40 x10E9/L	4.25
Hemoglobin	13.1–16.8 g/dL	6.1
Platelets	180–397 x10E9/L	263
High-sensitive C-reactive protein	<=5.0 mg/L	44.9
Erythrocyte sedimentation rate	2–10 mm/h	44
Sodium, serum	135–145 mmol/L	139
Potassium, serum	3.5–5.2 mmol/L	4.2
Urea, serum	3.0–9.2 mmol/L	4.4
Creatinine, serum	60–110 umol/L	71
Vitamin B12	138–652 pmol/L	221
Folate	7.0–46.4 nmol/L	10.1
Syphilis screen		Non-reactive (Treponemal antibody not detected)
HIV antigen–antibody		Non-reactive
PCOT glucose	Random: 4.0–7.8 mmol/L	7.0 mmol/L
Cerebrospinal fluid		
White blood cell count		< 1/μL
Red blood cell count		3/μL
Protein	0.15–0.40 g/L	0.29 g/L
Glucose	2.2–3.9 mmol/L	3.9
Meningitis and Encephalitis DNA and RNA PCR	The detectable pathogen types are listed as follows: *Escherichia coli* K1, *Haemophilus influenzae*, *Listeria monocytogenes*, *Neisseria meningitidis*, *Streptococcus agalactiae*, *Streptococcus pneumoniae*, Cytomegalovirus, Enterovirus, Herpes simplex virus 1, Herpes simplex virus 2, Human herpesvirus 6, Human parechovirus, Varicella zoster virus, *Cryptococcus neoformans*/gattii.	Not detected
RT-QuIC		Positive
T-tau protein	0–1,149 pg./mL	> 20,000 pg./mL
14–3-3 Gamma	<30–1999 AU/mL	69,601
Montreal cognitive assessment		13 /30 Executive: 1/5 Naming: 3/3 Attention: 3/6 Language: 1/3 Abstraction 1/2 Delayed recall: 0/5 Orientation: 4/6
Electroencephalography		Right frontotemporal epileptogenic focus with intermittent discharges at a rate of 1 Hz

**Figure 2:**
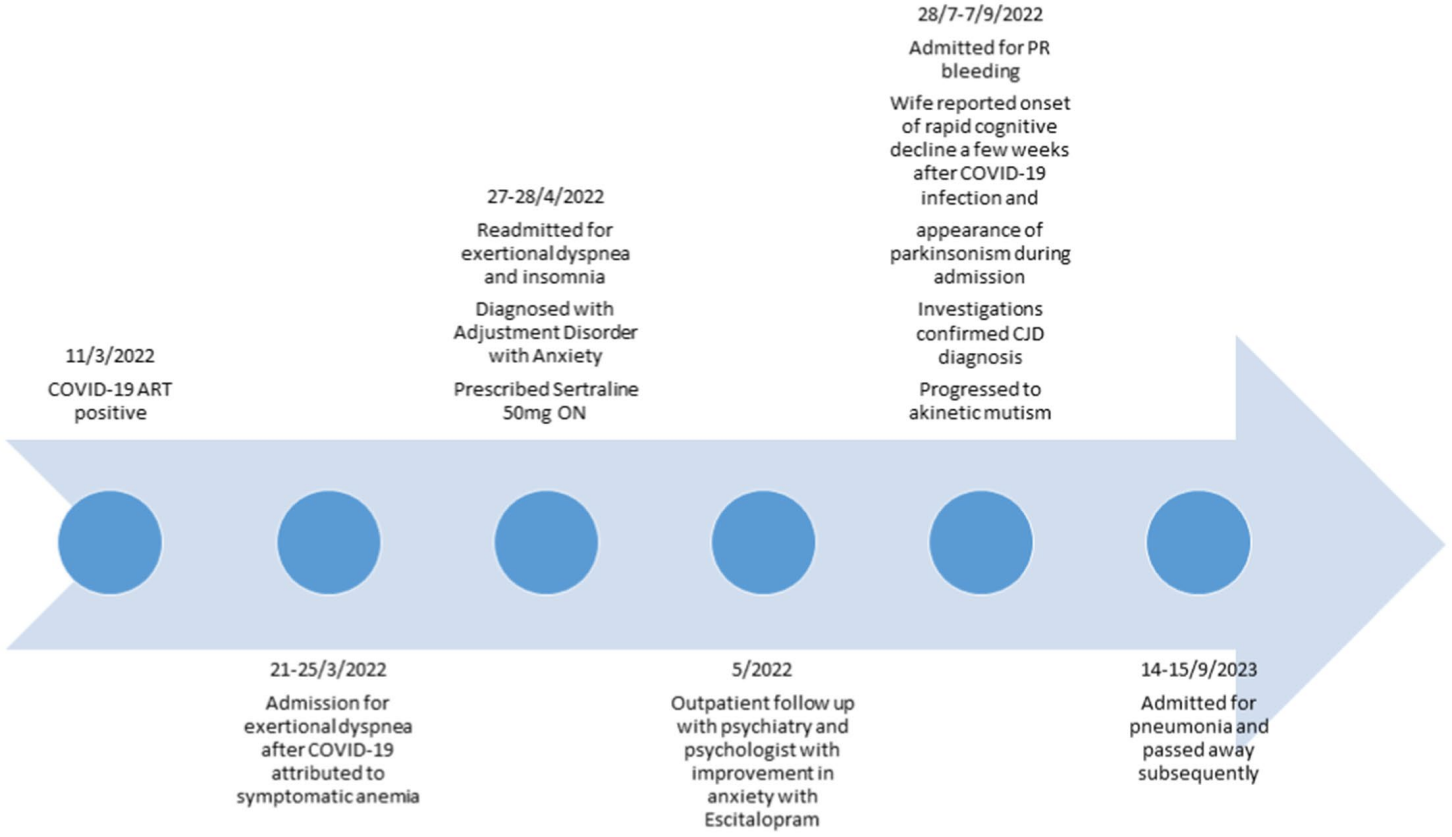
Timeline of clinical course.

His condition progressed with worsening confusion, disorientation and inability to recognize the medical staff. He remained mostly cheerful without significant episodes of anxiety or agitation. His processing speed slowed further, resulting in a paucity of verbal replies. He also developed worsening bradykinesia and myoclonus. The patient passed away from pneumonia soon after his hospital discharge. This was 1 month from the diagnosis of CJD and 5 months following his COVID-19 infection (Figure 2).

## Discussion

There have been several case reports citing the onset of CJD following COVID-19 infection. These cases presented at the early stage with mostly neurological symptoms (6–8). We report a patient presenting after a mild COVID-19 infection with initial prominent anxiety symptoms followed by rapid cognitive decline. The clinical suspicion for and eventual diagnosis of sporadic CJD was challenging as the patient’s presenting anxiety symptoms were initially attributed to multiple somatic complaints and interpersonal conflicts. This was further confounded by a partial improvement in his psychiatric symptoms following antidepressant therapy. The subsequent report of cognitive complaints was attributed sequela following COVID-19 infection and not from a neurodegenerative disorder due to an absence of neurological deficits. However, the later emergence of symptoms of parkinsonism resulted in neuroimaging and CSF analysis which confirmed the diagnosis.

Historically, psychiatric symptoms are more common in the variant form of CJD (vCJD) than the sporadic form of CJD (sCJD) (9). In one study, symptoms of mood disorder (irritability, anxiety, low mood) were noted in about 41.6% of patients with CJD (both variant and sporadic form) at any stage of the disease, with 44.5% of patients having behavioral and psychiatric symptoms as part of their first symptoms of the disease (2). Another study showed that 30% of patients with CJD experienced anxiety, with 77% of them experiencing symptoms within the first 100 days of illness. They were often observed as anxious and fearful without obvious external triggers (10). Like our patient, it is not uncommon for patients with sCJD to manifest anxiety symptoms early in the illness.

The prion protein (PrPc) is a glycoprotein mostly found on cell membranes in the neurons of the CNS. CJD is caused by transforming PrPc into misfolded prion PrP scrapie (PrPSc), which is infectious and accumulates throughout the brain (11). While age and methionine homozygosity at Codon 129 of the prion protein gene are known risk factors for the development of CJD, the triggers for development of this disease are unknown (3).

SARS-CoV-2 invades the CNS directly through the olfactory epithelium or hematogenous route. This results in damage to the blood brain barrier (BBB), causing neuroinflammation. Systemic inflammation due to SARS-CoV-2 infection and activation of the immune system releases inflammatory cytokines and results in further breakdown of the BBB and exacerbates neuroinflammation (4). Similarly, neurodegenerative disorders such as CJD display an increased inflammatory response in the brain induced by PrPSc. This is evidenced by the presence of significantly raised markers of inflammation such as TNF-α, IL-1β, and IL-1α in the brain tissue of patients with CJD (12). Hypothetically, SARS-CoV-2 infection may trigger the pathogenic development of neurodegenerative disorders such as CJD via a neuroinflammatory process.

Microglia are the resident immune cells of the CNS and play a crucial role in maintaining CNS homoeostasis and regulation of inflammatory processes in the brain (13). Astrocytes regulate neurovascular function such as blood flow and permeability of the BBB (14). Some studies show that microglia activation is involved in the pathogenesis of CJD (13, 15). Microglia are initially activated by PrPSc and drawn to areas in the brain with accumulation of PrPSc to aid in its clearance (16). They take on a phagocytic role, producing anti-inflammatory cytokines such as TGF-β, IL-4 and IL-10. However, over time microglial clearance of prion proteins is insufficient. Accumulation of PrPSc results in damage to neurons, which trigger microglia toward a pro-inflammatory role, secreting pro-inflammatory cytokines such as IL-6, TNF-α and IL-1β, which lead to disease progression (13, 15). In the context of COVID-19, microglia and astrocytes respond to the proinflammatory signals from endothelial cells, macrophages, and neurons (17). Their activation increases the production and release of pro-inflammatory cytokines. This disruption of their homeostatic role induces neuroinflammation and neurodegeneration (14). This risk is increased in older adults as they often display increased microglia with dystrophic morphologies (18). Thus, given that microglia act as a cellular inflammatory mediator in prion disease and COVID-19, SARS-CoV-2 induced proinflammatory microglia may trigger and accelerate the progression of prion disease.

Furthermore, multiple reports have linked the ‘S1’ spike protein, found on the surface of SARS-CoV-2 (19), with prion diseases. The S1 spike proteins have been found to interact with heparin and heparin-binding protein, leading to the aggregation of various proteins in the brain, including Aβ peptides, α-synuclein, tau and prion proteins. These aggregations can potentially worsen inflammation, neurodegeneration and cell death in the CNS, resembling changes seen in prion diseases and/or Alzheimer’s disease (20, 21). Moreover, the ‘S1’ spike protein contains ‘prion-like’ domains that may play a role in systemic amyloidosis and systemic inflammation, causing neuroinflammation in the CNS and possibly of COVID-19 contributing to CJD (22).

## Conclusion

This case report adds to the literature suggesting that COVID-19 may potentially unmask the onset of CJD through the ‘S1’ spike protein promoting aggregation of prion proteins and SARS-CoV-2 induced pro-inflammatory microglial phenotype leading to neuroinflammation. However, this temporal relationship may be coincidental rather than a risk factor, so further studies are needed to evaluate the strength of the association. Nevertheless, it would be prudent to closely monitor patients who develop psychiatric symptoms and cognitive decline post-COVID-19 infection, as it may signal the emergence of an underlying neurodegenerative process.

## Data availability statement

The original contributions presented in the study are included in the article/supplementary material, further inquiries can be directed to the corresponding author.

## Ethics statement

Written informed consent was obtained from the individual’s next of kin for the publication of any potentially identifiable images or data included in this article.

## Author contributions

CC and CH contributed to the conception of the study. CY wrote the first draft of the manuscript. CY and EM performed a literature review. JL perused the MRI images, selected and labeled them. CY and EM wrote sections of the manuscript. All authors contributed to manuscript revision, read, and approved the submitted version.

## Conflict of interest

The authors declare that the research was conducted in the absence of any commercial or financial relationships that could be construed as a potential conflict of interest.

## Publisher’s note

All claims expressed in this article are solely those of the authors and do not necessarily represent those of their affiliated organizations, or those of the publisher, the editors and the reviewers. Any product that may be evaluated in this article, or claim that may be made by its manufacturer, is not guaranteed or endorsed by the publisher.
